# Book Reviews

**Published:** 1907-02

**Authors:** 


### BOOK REVIEWS.

*PETERSON'S OBSTETRICS.* The Practice of Obstetrics. By Eminent Authorities. Edited by Reuben Peterson, A. B., M. D., Professor of Obstetrics and Diseases of Women in the University of Michigan, Department of Medicine and Surgery, Ann Arbor, Mich. Large octavo, about 1087 pages, with 523 engravings and 30 full-page plates in colors and monochrome. Cloth, $6.00, net; leather, $7.00, net; half morocco, $8.00, net. Lea Brothers & Co., Philadelphia and New York, 1907.

With the volume edited by Prof. Reuben Peterson, The Practitioner's Library of Gynecology, Obstetrics and Pediatrics is completed. It is fully up to the high standard set by its companions, the Gynecology, edited by Bovee, and the Pediatrics, by Carr. The profession now has at command in convenient form an authoritative exposition of the latest and best knowledge upon three closely interrelated and important specialties. The basic subjects of applied pathology and etiology are considered with sufficient fulnesss to lay the foundation necessary for a fruitful understanding of the practical aspects, to which major space is devoted. Each author has woven in his own observations of disease and the therapeutic measures which have resulted in the greatest success. This adds to each chapter a personal element of obvious value. In view of their particular importance in Obstetrics, the series of illustrations has been made exceptionally rich, and it is likewise notable for being largely from original photographs taken from life. The facilities at Dr. Peterson's command have rendered it possible to an unusual degree to make selections specifically representing points in the text. Though it is to the interest of every practitioner to have this well-rounded and compact Library at hand, the volumes are sold either together or separately.

*STARR ON NERVOUS DISEASES.* Organic and Functional Nervous Diseases. By M. Allen Starr, M. D., Ph. D., LL. D., Professor of Neurology in the College of Physicians and Surgeons, New York; ex-President of the American Neurological Association and of the New York Neurological Society. Second edition, thoroughly revised. Octavo, 824 pages, with 282 engravings and 26 full-page plates. Cloth, $6.00, net; leather, $7.00, net. Lea Brothers & Co., Philadelphia and New York, 1907.

The author's position in the forefront of neurologists has been shown anew in the rapid exhaustion of the first edition of his work, limited though it was to organiz nervous diseases. An even warmer reception is assured for this revision, which brings the organic portion to date and adds a section covering the functional diseases, so that the volume now presents the whole field of neurology as understood and practised by a master. The author is the reverse of abstruse or nihilistic. On the contrary, he is straightforward and direct, and justifies his optimism as to the advanced position of neurological diagnosis and treatment by the wealth of information placed at command of his readers. Paying due regard to theory, he devotes especially full attention to etiology, diagnosis and treatment, both medical and surgical. The book is largely based on the solid foundation of long experience, but it also embodies the well-attested knowledge of other authorities as gleaned from a thorough sifting of the vast literature of neurology. Practical, authoritative, covering the whole subject in all its aspects, and abundantly illustrated, this new edition of Prof. Starr's work answers the needs of students, practitioners and specialists.

---

*PROGRESSIVE MEDICINE, VOL. IV., DECEMBER,* 1906. A Quarterly Digest of Advances, Discoveries and Improvements in the Medical and Surgical Sciences. Edited by Hobart Amory Hare, M. D., Professor of Therapeutics and Materia Medica in the Jefferson Medical College of Philadelphia. Octavo, 349 pages, with 29 engravings. Per annum, in four cloth-bound volumes, $9.00; in paper binding, $6.00, carriage paid to any address. Lea Brothers & Co., Publishers, Philadelphia and New York.

The December issue of this quarterly digest completes the series for 1906. It will be found a fitting climax to the thorough work which preceded it. The skilled contributors have finished their labors for the year with never failing energy and care. As a result the busy practitioner can cull in an hour what it has taken investigators and teachers the world over months, if not years, to acquire. Special stress has been laid on practical results and the best methods of treatment. Thus the chaff has been eliminated, and only the wheat remains. Dr. J. Dutton Steele devotes one hundred and twenty pages to Diseases of the Digestive Tract and Allied Organs. His presentation of the subject, his attention to the minutest details and his clear deductions must prove suggestive to every reader.

Dr. William T. Belfield treats of Genito-urinary Diseases from Tuberculosis to the Irrigation and Drainage of the Seminal Duct and Vesicle. Under the Diagnosis of Renal Disease, Dr. Kapsammers' report of over twenty thousand autopsies in Austrian hospitals, during the last ten years, is quoted.

---

*WHITMAN'S ORTHOPEDIC SURGERY.* A Treatise on Orthopedic Surgery. By Royal Whitman, M. D., Instructor in Orthopedic Surgery in the College of Physicians and Surgeons, New York; Chief of Orthopedic Department in Vanderbilt Clinic, New York. Third edition, revised and enlarged. Octavo, 900 pages, with 554 illustrations, mostly original. Cloth, $5.50, net. Lea Brother & Co., Philadelphia and New York, 1907.

Orthopedic Surgery has hitherto been commonly considered as the most technical of specialties, and as being limited to the hands of the few who could devise and understand the cumbrous apparatus identified with the name. Modern methods have brought a new dispensation to the numerous and universally distributed class of sufferers from mechanical defects in their own bodies. Chief among these advances is the fact that much of this suffering is preventable, and as much curable by attention early in life, when structures are plastic and treatment is both easier and more efficacious. Hence the family physician has become the most important of all orthopedists, for it is he who has the first opportunity, and who is thereby under the highest obligation, to detect, prevent or cure such defects, or to recognize when they must be referred to a specialist. Dr. Whitman's book presents orthopedic surgery exactly along these modern lines, and the demand for successive large editions shows appreciation of its value by the general practitioner as well as the surgeon and specialist. The author has improved the opportunity again presented by the popularity of this standard book by thoroughly revising it to the latest date and incorporating new material and many new illustrations.

---

*TUTTLE ON DISEASES OF CHILDREN.* A Pocket Text-Book of Diseases of Children. By George M Tuttle, M. D., Attending Physician to St. Luke's Hospital, the Martha Parsons Hospital for Children and Bethesda Foundling Asylum, St. Louis, Mo. New (2d) edition, thoroughly revised. In one 12mo volume of 392 pages, with 5 plates. Cloth, $1.50, net; flexible leather, $2.00, net. Lea's Series of Pocket Text-Books, edited by Bern. B. Gallaudet, M. D. Lea Brothers & Co., Philadelphia and New York, 1907.

The excellence of Prof. Tuttle's work has carried it through two printings and now to a new edition, revised to represent advances to date. By considering only those diseases and phases of disease which are peculiar to the period from birth to adolescence, the author has found space to present all the essentials of pediatrics within a very compact volume. The various diseases are systematically discussed under appropriate sub-headings, the typograhical arrangement being such that any desired point may be instantly found. The low price reflects the popularity of Lea's Pocket Text-Book Series, of which this volume is a constituent.

---

*PHYSIOLOGICAL CHEMISTRY IN THE SERVICE OF MEDICINE*, by Dr. Wolfgang Pauli, Privatdocent in Internal Medicine at the University of Vienna. Translated by Dr. Martin

H. Fisher, Professor of Pathology at the Oakland College of Medicine. 12mo, IX ? 156 pages. Cloth, $1.25, net. John Willy & Son, New York.

This work begins with the fundamental principles underlying modern chemistry in order for its readers to obtain a conception of its applicability to biological and physiological questions. It comprises seven scholarly addresses by Dr. Pauli, which have been excellently translated by Dr. Fisher.

Pathology has developed much more rapidly toward the morphological than towards the chemical side, and the work done shows that we have only begun to solve the many important questions before us. The work done by Dr. Pauli is to be much commended, and all who care to think deeply upon these subjects should study this book, for it will enable them to perceive a large number of phenomena now hidden.

---

*PLASTER OF PARIS AND HOW TO USE IT*, by Martin W. Ware, M.D., Adjunct Attending Surgeon, Mount Sinai Hospital; Surgeon to the Good Samaritan Dispensary; Instructor in Surgery, N. Y. Post Graduate Medical School. 12mo; 72 illustrations, about 100 pages. Surgery Pub. Co., 92 William St., New York City. Cloth, $1.00.

This is one of the most useful books ever presented, not only on account of the general demand for the information and instructions upon the subject which this book so explicitly, practically and comprehensively covers, but because this knowledge was not previously available except from such a vast experience as enjoyed by Wr. Ware, or, in part, by reference to many books on allied subjects.

It is a vivid narrative, profusely illustrated, of the many uses to which Plaster of Paris is adaptable in Surgery. The whole subject, from the making of the Bandage to its use as a support in every form of splint, corset or dressing, is graphically described and illustrated. The use of Plaster of Paris in Dental Surgery is also covered. The book is presented in the artistic manner characteristic of the productions of the Surgery Publishing Company. It is printed upon coated book paper and attractively bound in heavy red buckrum, stamped in white leaf and gold. Price $1.00.

---

*DISEASES OF CHILDREN*, By John Ruhrah, M. D,. W. B. Sanders & Co., publishers. Price, $2.50, net.

Dr. Ruhrah's book is intended chiefly for students, but will be found equally useful for practitioners. The work is clear, sufficiently full and well up to date. It will be a worthy addition to pediatrics.

---

*ATLAS AND TEXT-BOOK OF HUMAN ANATOMY.* Volume II. By Professor J. Sobotta, of Wurzburg. Edited, with additions, by J. Playfair McMurrich, A. M., Ph. D., Professor of Anatomy at the University of Michigan, Ann Arbor. Quarto volume of 194 pages, containing 214 illustrations, mostly all in colors. Philadelphia and London: W. B. Saunders Company, 1906. Cloth, $6.00 net; Half Morocco, $7.00 net.

This entirely new work on human anatomy is undoubtedly the most beautiful and practical ever produced, the numerous large quarto-size plates representing in colors the anatomic structures with wonderful accuracy and clearness. Professor Sobotta's concise, yet clear style, so admirably maintained in the English translation, makes the work an ideal text-book for the student and an invaluable aid to the physician, surgeon, and anatomist. Volume I. treats of bones, ligaments, joints, and muscles; volume II. of the viscera, including the heart; volume III. of the nervous and circulatory systems, and the organs of the senses. Three quarto volumes, each containing 250 pages of text and over 300 illustrations, mostly in colors. By Prof. J. Sobotta, of Wurzburg. Edited, with additions, by J. Playfair McMurrich, A. M., Ph. D., Professor of Anatomy, University of Michigan. Per volue: cloth, $6.00 net.

---

*A MANUAL OF PATHOLOGY.* By Guthrie McConnell, M. D., Pathologist to the St. Louis Skin and Cancer Hospital and to St. Luke's Hospital, St. Louis, Missouri. 12mo of 523 pages, illustrated. Philadelphia and London: W. B. Saunders Company, 1906. Flexible leather, $2.50 net.

Dr. McConnell's manual of pathology treats the subject from the clinical point of view. It is a pathology for the general practitioner and student, and therefore disputed theories and controversial subjects have been omitted. A further object was to present his subject in as concise a manner as possible, making the work serviceable as a quick reference book for the busy practitioner. Illustrations have been most freely introduced, aiding greatly in the recognition of diseases. There is a large number of microscopic pictures illustrating pathologic conditions which will be found especially valuable to the general practitioner. Dr. McConnell's hospital connections have furnished him with excellent material for the laboratory study.

---

*THE ELEMENTS OF THE SCIENCE OF NUTRITION.* By Graham Lusk, Ph.D., M.A., F.R.R. (Edin.), Professor of Physiology at the University and Bellevue Hospital Medical College, New York City. Octavo of 326 pages, illustrated. Philadelphia and London: W. B. Saunders Company, 1906. Cloth, $2.50 net.

The appearance of a work on nutrition at this time is indeed opportune, for the subject of nutrition is now receiving the widespread attention that its importance demands. Dr. Lusk in this new work reviews in as concise a manner as possible the scientific foundation upon which rests our knowledge of nutrition. His constant aim has been to make his book useful alike to the student and general practitioner, and he has treated his subject in its relation to both health and disease. Every statement is the direct result of actual experience, so that the work is eminently practical as well as scientifically accurate. The general practitioner, therefore, in his daily practice, will find this work of the greatest value.

---

*A TEXT-BOOK OF DISEASES OF WOMEN.* By J. Clarence Webster, M. D. (Edin.) F. R. C. P. E., F. R. S. E., Professor of Obstetrics and Gynecology in Rush Medical College, in affiliation with the University of Chicago. Large octavo of 712 pages, with 372 text-illustrations and 10 colored plates. Philadelphia and London: W. B. Saunders Company, 1907. Cloth, $7.00 net; Half Morocco, $8.00 net.

Dr. Webster's work on diseases of women is based on his extended clinical experience, and unusual prominence is given to the scientific basis of each subject under consideration. Special endeavor has been made to include all the important original investigations of recent years, so that the work represents the present-day knowledge upon a subject of the greatest importance to every practitioner. Indeed, Dr. Webster has written this work especially for the general practitioner, discussing the clinical features of the subject in their widest relations to general practice rather than from the standpoint of specialism. The magnificent illustrations, some four hundred and fifty in number, are nearly all original. Drawn by leading medical artists under Dr. Webster's direct supervision, they portray the anatomy of the parts and steps in the operations with rare clearness. The general practitioner will find it just the work he has long needed.

---

*RYMOTHERAPY.* A Discussion of the Physiological Basis of the Therapeutic Potency of Mechano-Vital Vibration, to Which is Added a Dictionary of Diseases with Suggestions as to the Technique of Vibratory Therapeutics, with Illustrations by Samuel S. Wallian, A. M., M. D. Chicago, Ill.: The Ouellette Press, 1906.

This book deals with a subject of increasing interest to the profession. It is an excellent guide to the use of vibratory therapeutics, and as such will be welcomed by all progressive physicians.

---

*A TEXT-BOOK UPON THE PATHOGENIC BACTERIA.* For Students of Medicine and Physicians. By Joseph McFarland, M. D., Professor of Pathology and Bacteriology in the Medico-Chirurgical College, Philadelphia. New (5th) Edition. Octavo volume of 647 pages, fully illustrated, a number of colors. Philadelphia and London: W. B. Saunders Company, 1906. Cloth, $3.50 net.

This entirely new work is a plain account of the natural history of disease, covering the field completely. Of the work, American Medicine says: "We feel confident in saying no other treatise, not encyclopedic in character, on any subject contains so much direct and correlated information on the branch with which it deals." Unlike most works on Pathology, it treats the subject not from the professor's standpoint, but from that of the student, the author ever aiming to render most easy of comprehension the many difficult theories of the science. The work is descriptive, not controversial, the debated points usually not being discussed. The text is profusely illustrated with practical illustrations, a number of them being in colors and printed directly in the text.

---

In the treatment of hand and finger infections, it is very important to release from bandaging as much and as many of the fingers as possible, and as soon as possible. The habit of bandaging up immovably all the fingers, in the treatment of a lesion of some of them, saves the surgeon time but, except in short cases, it often cripples the hand by stiffening the fingers.—*American Journal of Surgery.*

---

Carcinoma of the prostate often does not recur for some time; meantime the patient may look surprisingly well. This should not beguile the surgeon into a too hopeful prognosis.—*American Journal of Surgery.*

---

If a bubo shows no sign of disappearing under wet dressings, ice bags, etc., and evidences of suppuration are developing, it is better to make a clean dissection and excise the gland without opening it than to incise and drain.—*American Journal of Surgery.*

---

In cases of [illegible] colic do not make too positive a pre-operative diagnosis of calcus, no matter hw typical the syptoms may be. It has happened very often at the time of operation that no stone is found. Fortunately, these cases are nearly always cured by the expioratory nephrotomy.—*American Journal of Surgery.*

---

The surgeon hould not wait for redness before making a diagnosis of palmor abscess. Owing to the density of the fascial structures this sign is often lacking in the early stages.—*American Journal of Surgery.*

